# Partial molar pregnancy with a normal live fetus and umbilical cord abnormalities: A novel association with long‐term follow‐up: A case report

**DOI:** 10.1002/ccr3.4839

**Published:** 2021-09-15

**Authors:** Seyedeh Noushin Ghalandarpoor Attar, Seyedeh Mojgan Ghalandarpoor Attar

**Affiliations:** ^1^ Obstetrics and Gynecology Department Imam Khomeini Hospital Tehran University of Medical Sciences Tehran Iran; ^2^ Obstetrics and Gynecology Department Baharloo Hospital Tehran University of Medical Sciences Tehran Iran

**Keywords:** partial mole, umbilical cord cyst, umbilical cord varix

## Abstract

A euploid fetus in a partial molar pregnancy can develop umbilical cord abnormalities as pregnancy goes on. So, careful examination of the umbilical cord can determine fetuses at risk for ominous adverse effects.

## Graphical abstract

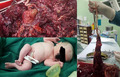

## 1 INTRODUCTION

A partial molar pregnancy with a normal live fetus is extremely rare and reports of long‐term follow‐up are lacking. Here, we present a case of such pregnancy with an extremely rare umbilical cord abnormality, which led to fetal distress and pregnancy termination. This association seems to be novel.

The incidence of a partial molar pregnancy with a live fetus is approximately 0.005–0.01 in all pregnancies, and the incidence of a normal live fetus is even rarer.^1^, ^2^ Survival of the fetus depends on fetal karyotypes, abnormal molar placental mass size, and rate of molar tissue degeneration, indicating fetal anemia or other obstetric complications.^3^

Molar pregnancy may occur as a singleton pregnancy or as part of a dichorionic diamniotic twin pregnancy. Such twin pregnancies (a normal fetus with a complete mole) are predisposed to more adverse events, such as spontaneous abortion, preeclampsia, thromboembolism, vaginal bleeding, still birth, preterm labor, invasive mole, or choriocarcinoma, and probable hysterectomy.^4^ In the majority of cases, these adverse events can lead to pregnancy termination. In this regard, Fishman et al^5^. reported an incidence of up to 73% of pregnancy termination. On the other hand, Sebire et al^6^. reported the incidence of live birth and gestational trophoblastic neoplasia (GTN) to be 40%.

If there is a euploid fetus in a singleton partial molar pregnancy, the placenta can have a variety of karyotypes ranging from diploid amnion cells to triploid chorionic villi.^7^ Although continuing pregnancy is possible, couples should be fully consulted about the mentioned adverse events. Most pregnancies, which include a normal live fetus in association with molar changes, are twin gestations.^2^ Due to the rarity of such pregnancies, management is challenging and debated.

Here, we discuss a partial molar pregnancy with a euploid normal fetus in the presence of an umbilical cord cyst, concomitant with an extra‐abdominal umbilical vein varix, all of which are very rare cord abnormalities by themselves. Our management resulted in the birth of a healthy neonate, who showed no neurodevelopmental delays in the 2‐year follow‐up.

## 2 CASE REPORT

A 29‐year‐old primiparous woman at the gestational age of 23 weeks was referred to our clinic. Her first‐trimester aneuploidy screening had suggested an intermediate risk, and the subsequent sequential screening for trisomy 21 and neural tube defects (NTD) had shown high‐risk results. Amniocentesis was performed, indicating a euploid female fetus. The amniotic fluid alpha‐fetoprotein (AFP) level was in the normal range (71.40 ng/ml or 2.42 MoM). She had been strongly suggested a detailed anomaly scan, and a thick cystic appearance placenta in favor of a partial molar pregnancy was found. After a comprehensive consultation and ruling out a metastatic condition, she decided to continue her pregnancy despite possible serious adverse events. Next, her beta‐HCG was measured serially, which was found to decrease from 172,000 at week 15 of gestation to 68,000 ng/ml at week 32 of gestation.

**FIGURE 1:**
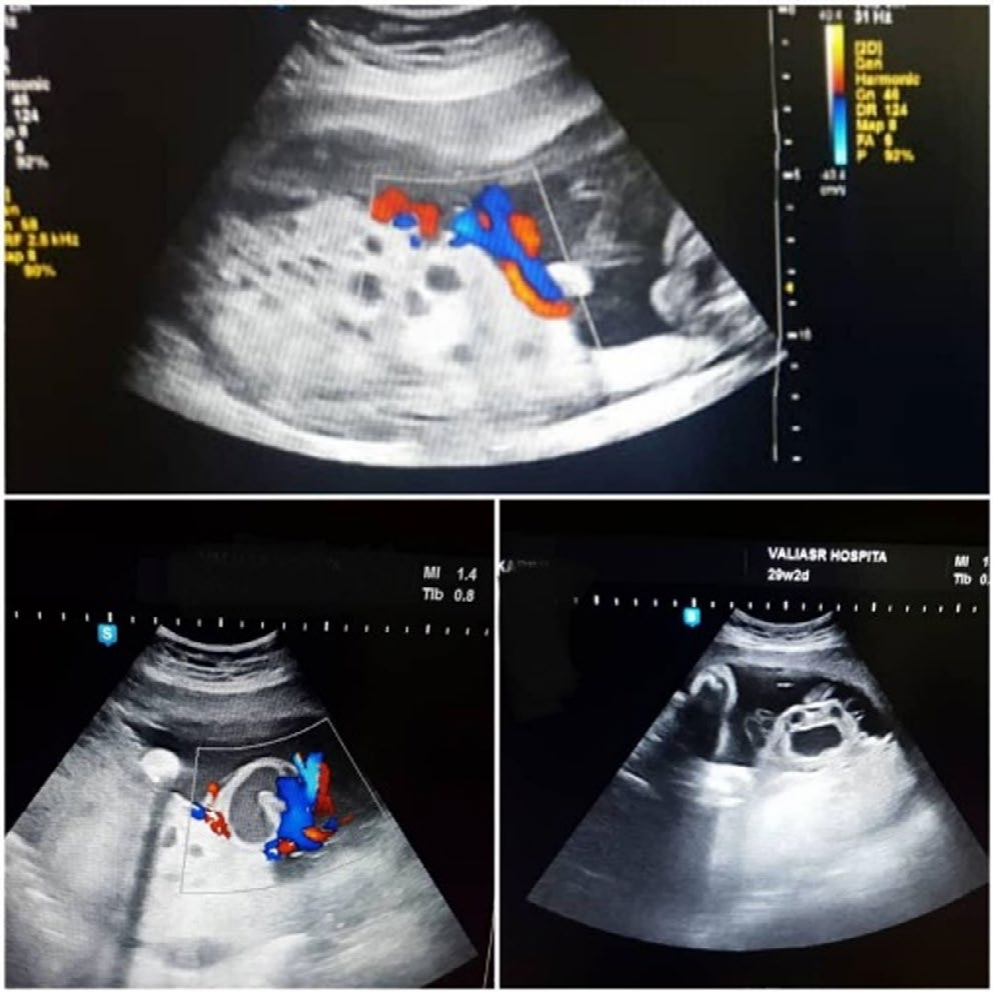
Upper image shows cystic appearance of the placenta and lower image shows the varix of umbilical vein in color Doppler (right side) and Gray scale Imaging (left side)

The second‐targeted ultrasound conducted at 23 weeks of gestation was similar to the previous one. However, at 29 weeks of gestation, we found an umbilical vein varix at the placental site of umbilical cord insertion (measuring 43 × 50 mm), as well as a true cord cyst (28 × 42 mm; Figure 1). Subsequently, we arranged 2 weekly antenatal fetal surveillance tests. The fetal growth curve was normal, and the placenta and the umbilical vein varix were stable in size and appearance until the last examination. The patient remained normotensive and euthyroid during pregnancy.

**FIGURE 2:**
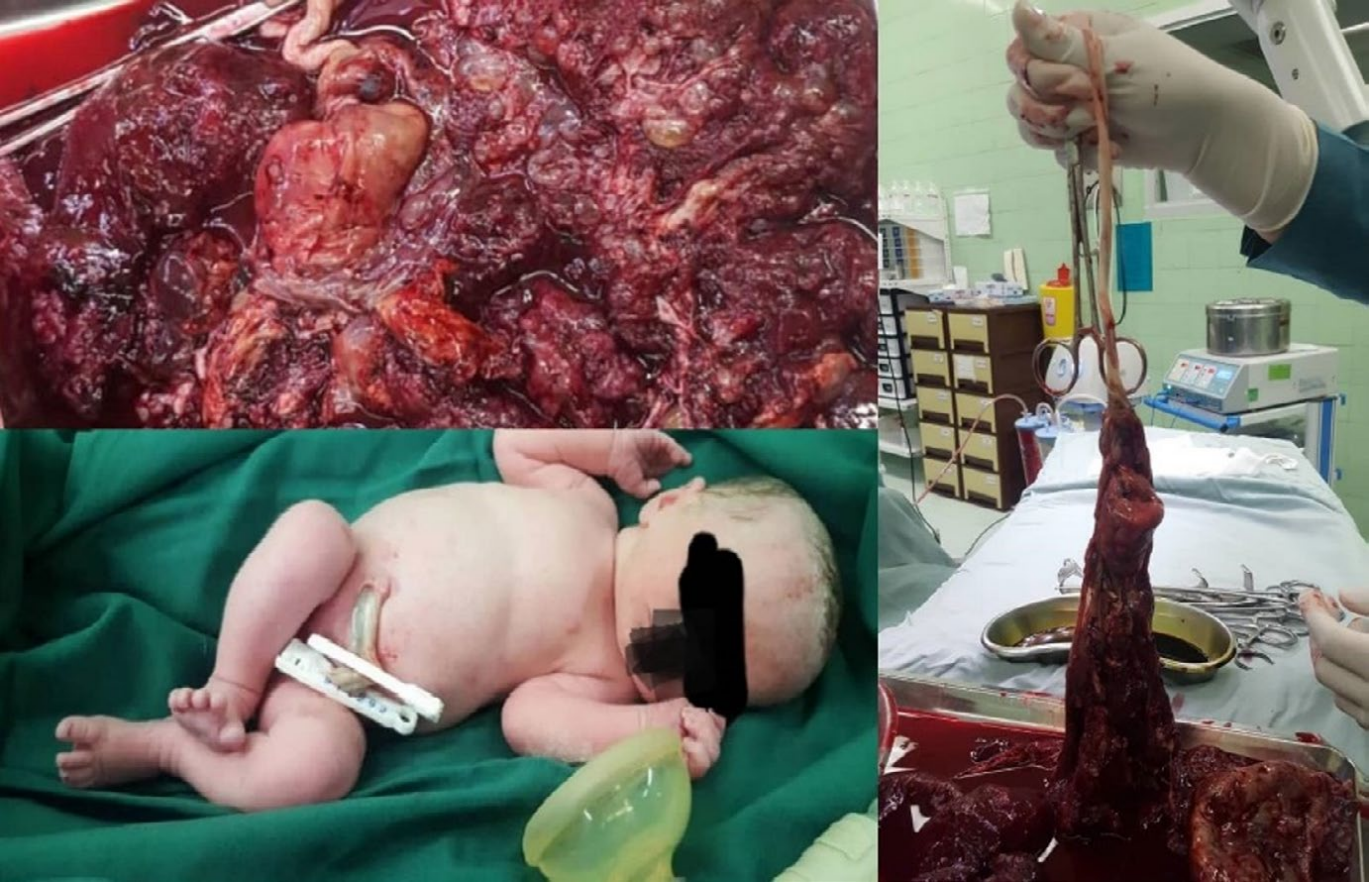
It shows gross appearance of placenta (upper right side Image) and umbilical cord (left side) and the normal appearance of newborn (lower right side)

**FIGURE 3:**
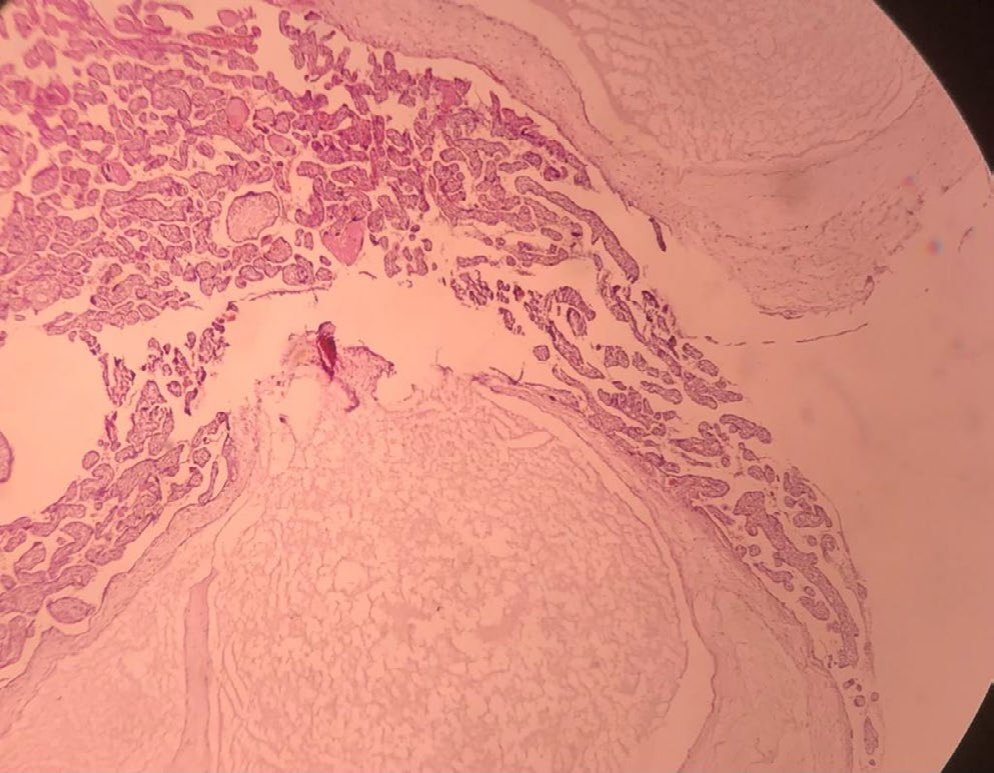
Histopathologic examination of the placenta showed molar changes

Finally, at 34 weeks of gestation, the patient visited our center with a complaint of decreased fetal movements. Fetal heart monitoring showed intermittent late decelerations in conjunction with decreased beat‐to‐beat variability. She underwent an emergency cesarean section, and a female neonate weighing 1980 g, with 1‐ and 5‐min Apgar scores of seven and nine, respectively, was delivered. The umbilical cord pH and the base deficit were 7.33 and −3.8 mmol/L, respectively. After delivery, gross examination of the placenta revealed multiple vesicular lesions; an umbilical vein varix and a true cyst were confirmed too. The varix was also complicated by the presence of thrombosis and clot formation (Figure 2). Molar changes were also confirmed by histologic examination (Figure 3).

It is worth mentioning that in the nursery ward, the neonate experienced hypoglycemia. She was breastfed, but because of persistent hypoglycemia, respiratory distress, and pallor, she was admitted to the NICU. She also developed generalized petechiae in the NICU. The initial laboratory tests are shown in Table 1. In the following days, the daily platelet count increased, reaching 194,000/mm^3^ on the sixth day. The brain MRI showed decreased sulci and thinning of the corpus callosum, besides posterior horn dilation of lateral ventricles, all of which disappeared spontaneously in the follow‐up visits. The mother's serum beta‐HCG decreased to 11,000 in the 24‐h postpartum visit weekly follow‐ups beta‐HCG level became undetectable one month after delivery. The neonate was also followed monthly for 6 months, then every 3 months for a year, and twice a year in the second year. At the time of writing this paper, the patient was completely healthy, there was no GTN, and her neonate had normal growth and development.

**TABLE 1:** The neonate's laboratory tests

Test	Result	Test	Result
WBC	30.2*10^3^/mm^3^	Umbilical cord PH	7.23
RBC	3.52*10^6^/cumm	Umbilical cord HCO3	21.8 mmol/L
Hb	13 gr/dl	Umbilical cord BE	−6.3 mmol/L
Hematocrit	41.2%	Umbilical cord PCO2	51.5 mmHg
MCV	117 FL	Blood sugar	20 mg/dl
MCH	36.9 Pg	Blood group	B positive
MCHC	31.6%	Direct coombs	Negative
Plt	60*10^3/mm3	G6PD	Sufficient
Reticulocytes	280000 cell/mm3	Blood culture	Negative
Direct bilirubin	0.5 mg/dl	Creatinine	0.6 mg/dl
Total bilirubin	5.9 mg/dl	TORCH study	Negative

## 3 DISCUSSION

We presented a confirmed case of partial molar pregnancy with a normal live fetus. Two detailed sonographic examinations at weeks 18 and 23 of gestation did not indicate any major structural anomalies. However, a small true umbilical cyst (measuring 43 × 25 mm), as well as an extra‐abdominal intra‐amniotic varix of the umbilical vein (measuring 51 × 42 mm), was documented in the ultrasound conducted at 29 weeks of gestation. It should be noted that such pregnancies are extremely rare, and there are few case reports and case series in the literature.^1^, ^2^

Besides this rare pregnancy, our patient had two minor umbilical anomalies. It is known that umbilical cord anomalies comprise 4% of all fetal malformations, and the prevalence of umbilical vein varix is estimated at 0.4–1.1 in 1000; extra‐abdominal varices are even rarer than the intra‐abdominal ones.^8^, ^9^ Cord varices are often diagnosed between 22 and 33 weeks of pregnancy in women with usually normal previous sonographic examination.^10^ Some believe that these anomalies are developmental rather than congenital due to increased intraluminal pressure.^10^

In our literature review, we could not find any similar case reports, and our case seems to be the first report of the co‐occurrence of a partial molar pregnancy (with a normal euploid fetus) with umbilical cord abnormalities. Our case indicates the importance of assessing the umbilical cord carefully in these patients. However, detailed examination of the umbilical cord using high‐resolution ultrasonography can be difficult, and most physicians do not have enough experience.^11^

On the other hand, since it is believed that umbilical vein varices are developmental abnormalities, congested circulation in the placenta and the cord in a molar pregnancy may explain the varix formation, as seen in our case. Interestingly, our fetus was female, which is consistent with a reported predominance of female fetuses to males in the literature.^12^ Our patient also had an increased risk of NTD and an elevated AFP level (2.9 MoM) in her second trimester. Feirburg et al^1^. suggested that an elevated AFP level could be a diagnostic marker for a molar pregnancy. Moreover, Hsieh et al^2^. reported a case of a partial molar pregnancy with an increased level of AFP. An explanation for this finding can be the subchorionic or intra‐amniotic hemorrhage due to the possibility of ruptured hemorrhagic vesicles in molar placentas.

Another interesting point in our case is that pregnancy was terminated due to suspected fetal distress. Considering the presence of an approximately large umbilical vein varix complicated by thrombosis, this complication may be the initial cause of fetal distress. Besides, thrombosis formation in the varix can be a reason for our newborn thrombocytopenia and anemia at birth. Although in the presence of umbilical vein varices, many physicians still recommend preterm pregnancy termination as soon as lung maturation is documented,^13^ recent studies suggest close follow‐up and continued pregnancy until term.^14^

In the present case, we found the concomitant incidence of a partial molar pregnancy and an umbilical vein varix in our patient, and to the best of our knowledge, this is the first case. Our patient had not experienced any complications due to molar pregnancy until delivery. It seems that thrombosis formation in the varix only had caused fetal distress, which had led to an emergency cesarean section at 34 weeks of gestation; however, the neonatal outcome was promising. Therefore, in similar cases, physicians must pay more attention to the delivery time.

In our literature review, we could only find very rare cases of partial molar pregnancy with long‐term neonatal outcomes; our case seems to be the first report of a long‐term follow‐up of a neonate (2 years). Except for transient thrombocytopenia and anemia immediately after birth, the brain MRI showed decreased cerebral sulci, thinning of the corpus callosum, and colpocephaly. Although umbilical vein varices have been reported in association with ventriculomegaly,^15^ it seems that cerebral changes in our neonate were a result of prematurity, as they disappeared in the follow‐ups, and the neurodevelopmental growth was normal.

## 4 CONCLUSION

The present case suggested that a partial molar pregnancy with a normal euploid fetus can result in the live birth of a healthy neonate. However, major attention must be paid to detailed structural anomalies in these fetuses, especially the most challenging ones, such as umbilical vein varices, by ultrasonography. Also, it seems that serial color Doppler sonography of umbilical vessels is reasonable for fetal surveillance to find any fetal or cord complications.

## CONFLICTS OF INTEREST

The authors declare that they have no competing interests.

## AUTHOR CONTRIBUTIONS

Seyedeh Mojgan Ghalandarpoorattar and Seyedeh Nooshin Ghalandarpoorattar made substantial contributions to conception, design, acquisition, and interpretation of data. They both were involved in drafting and revising the manuscript. Both authors read, corrected, and approved the final manuscript.

## ETHICAL APPROVAL AND CONSENT TO PARTICIPATE

The manuscript does not contain any direct patient identification details and hence ethics committee approval was waived.

## Data Availability

All relevant data is available at vali‐e‐asr Hospital, south Khorasan. Data sharing is not applicable to this article as no datasets were generated or analyzed during the study.
